# Lower back pain in young climbers: a retrospective cross-sectional study

**DOI:** 10.3389/fspor.2023.1328811

**Published:** 2023-12-22

**Authors:** Attilio Carraro, Barbara Gilic, Riccardo Bertolo, Andrea Albergoni, Fabio Sarto, Roberto Roklicer, Diego Sarto

**Affiliations:** ^1^ Faculty of Education, Free University of Bozen-Bolzano, Bozen, Italy; ^2^ Faculty of Kinesiology, University of Split, Split, Croatia; ^3^ Associazione Sportiva Dilettantistica Opera Verticale, Scorzè, Venezia, Italy; ^4^ Department of Biomedical Sciences, University of Padova, Padova, Italy; ^5^ School of Human Movement Sciences, University of Padova, Padova, Italy

**Keywords:** chronic injuries, injury surveillance, sport climbing, youth athletes, well-being

## Abstract

**Objective:**

The popularity of sport climbing has been growing since its inclusion in the Olympic Games program, which led to more people practicing it on recreational, amateur, and professional levels. Strenuous climbing training sessions and competitions might lead to frequent and serious musculoskeletal injuries and complaints among competitive climbers. This study aimed to investigate the prevalence of low back pain (LBP) and to explore the influence of various risk factors on LBP in adolescent climbers.

**Methods:**

The sample included 180 competitive climbers (46.6% males) aged 13–19 years competing in under-16 (48.3%) or under-20 categories. Data collection was carried out using the Nordic Musculoskeletal Questionnaire (NMQ) and the Graded Chronic Pain Scale (GCPS).

**Results:**

A total of 74.4% of the entire sample of participants (male = 75%; female = 74%) reported lower back complaints throughout the past twelve months, and only 15.5% during the last seven days. A major part of complaints was classified as low intensity-low disability (Grade I, 62.8%; male = 72.6%; female = 54.2%). Under-20 competitors reported a small but significantly higher percentage of almost all NMQ measures compared to under-16 athletes.

**Conclusions:**

This study found a relatively high prevalence of LBP, although complaints were of limited severity and did not affect climbers' regular training practice. Moreover, climbers did not differ in LBP prevalence according to sex, while climbers from the older age group reported higher complaints and seeking medical attention than younger climbers. Future studies should prospectively monitor the influence of climbing on LBP in youth climbers.

## Introduction

Climbing is a fast-growing sport. According to the International Federation of Sport Climbing (IFSC), 35 million climbers were estimated worldwide in 2015, while in 2018, the number rose to 44.5 million (IFSC 2018). Numerous studies have reported a variety of injuries as a result of climbing activities (1, 2). Climbing places extreme loads mostly on the upper extremities, followed by the lower extremities and the rest of the body, which means that no anatomic location is spared from climbing-related injury (2). The most prevalent injuries occur to the upper extremities, with fingers, elbows, and shoulders representing 80% of chronic injuries in climbing (1). Specifically, a study on 667 active climbers noted that two of three climbers had chronic injury, and the most prevalent injury sites were fingers (41.3%), shoulder (19.4%), and elbow (17.7%) (3). Moreover, the most common acute injuries are a result of a fall involved lower extremity (4).

Interestingly, gender differences concerning the injury site and injury prevalence have been reported, with the most frequent injury sites for females being fingers, shoulder, and wrist, and for males fingers, elbow, and shoulder (3). Moreover, one study on 1962 climbers from different countries aged 32.82 ± 9.4 years reported that females had a higher incidence of injuries compared to males, which was explained by the anatomical differences between sexes, e.g., male athletes reported twice the incidence of hand ligament injuries (5). Also, level of climbing experience and the overall training frequency have been reported as general predictors of injury in sports (i.e., a higher years of training experience and training hours led to a higher incidence of injuries), and similar was reported in climbers (6). The age at which climbers begin to compete has decreased during the last 10 years, meaning that adolescents are engaged in structured training programs which place enormous stress on the skeleton that is still immature (7, 8). Also, adolescents are more prone to injury due to adolescent growth spurts, differences in maturity status, and non-linearity of growth (7). Interestingly, older age (more than 15 years of age) and previous injuries were reported as one of the main risk factors in climbing, which means that adolescents should be monitored over time to try to prevent more serious injuries (9).

Most climbing-related injuries are from chronic overuse (19%–33%), acute atraumatic (28%), and 10%–39% are acute traumatic as a result of falls (5, 10). Thus, previous studies focused mainly on acute and chronic injuries, while musculoskeletal complaints are poorly studied in climbers. Musculoskeletal complaints are important because they are often underdiagnosed but can cause significant pain and reduced function (11). Low back pain (LBP) is one of the most common complaints worldwide (12). Indeed, it is estimated that more than 80% of the population would report LBP at some point during a lifetime (13). This represents a frequent disability condition leading to being unable to work and representing a social and economic burden (14). One of the most advocated preventions of LBP is physical activity (15). However, it has been proven that LBP and physical activity have a U-shaped relationship, meaning that too little or too much activity could be harmful to the health condition of the spine (14). Thus, as athletes are partaking in strenuous and vigorous-intensity physical activity, they are considered extremely susceptible to developing chronic LBP (16). Indeed, 10%–15% of all sports-related complaints are low back injuries and complaints (17). Moreover, adolescents have a greater risk of developing spinal problems as their musculoskeletal system is not mature and they are still undergoing excessive height growth (18). It has been reported that LBP is a common problem among children and adolescents (70%–80% lifetime prevalence before 20 years of age), and one of the risk factors for LBP in this age group is sports participation (19, 20). LBP among youth athletes should be taken seriously as it is usually associated to structural injuries such as spondylolysis and injuries to the posterior parts of the spine (21). Thus, LBP should be monitored and treated especially among young athletes who are still in the developmental life phase.

The impact of competitive climbing on LBP is debated; there are studies showing potential benefits but repetitive falls, unphysiological postures and very high lumbar muscles activation could represent potential risk factors. Namely, since climbing includes repetitive falls (i.e., falls on the mat during bouldering or in the rope during sport climbing), the stress which those falls put on the spine could be seen as potentially harmful (8). Moreover, other potential mechanisms are the prolonged high activation of paraspinal muscles and quadratus lumborum, and unphysiological postures that could lead to disc rotation and compression (22, 23). Indeed, there is a well-known postural dysfunction called “climbers back” which describes a postural adaptation of increased thoracic kyphosis which places the climber's body under unequally distributed forces on the musculoskeletal system (22). On the other side, a study on 30 patients aged 27.90 ± 6.08 years reported that climbing had a positive effect on LBP (24). However, this study involved a group of non-climbing people with LBP and a group of lower than amateur level participants. Previous research reported that only 5.3% of climbers had trunk pain which included LBP, making it less common than in the general (non-climbing) population (25, 26). However, as the climbing style changes due to the increased difficulty of the courses and a greater demand for spectacularism, the way of steep and three-dimensional wall architecture and dynamic moves (e.g., jumps that involve coordinated moves of feet and arms), injury patterns are changing correspondingly (8). Specifically, among 633 injuries within years 2017/18, there was a decrease in upper extremity injuries and increase of lower extremity injuries compared to studies observing periods of 1998–2001 and 2009–2012 (8).

Even though the most common injury sites in climbing are at the upper extremities, investigating other body parts in more detail is need, especially among younger populations, who are still in their growth and development phase (27). Thus, the main aim of this investigation was to determine the prevalence and severity (i.e., intensity and disability) of LBP in adolescent climbers. As sex, age, climbing volume, and years of experience are considered factors that influence higher susceptibility to injuries (6), the aim was also to investigate the influence of these factors on LBP in adolescent climbers.

## Materials and methods

### Study design

This study had a retrospective cross-sectional design, data were collected in sport clubs at the end of the competition season through online questionnaires. Participants completed the questionnaires independently and individually. The study rationale and informed consent formed the first pages of the package. Recruitment was carried out sending e-mail to sport clubs associated to the FASI (Italian Federation of Sport Climbing). Athletes were subsequently contacted via social networks or by telephone.

### Study population

Participants were members of climbing sport clubs from 15 regions in Italy. A total of 180 adolescent competitive climbers (84 males and 96 females), aged 13–19 years, participated in the study. They were divided into two age categories, U-16 and U-20, according to the International Federation of Sport Climbing (IFSC) rules 2023 Ver. no. 1.1 (IFSC rules 2023, https://cdn.ifsc-climbing.org/images/Website/2023_IFSC_Rules_112.pdf, Accessed on 10.7.2023.).

### Assessment tools

The questionnaire included three parts: (1) Demographic characteristics, training and competing background (questions related to the number of seasonal/daily competitions and training sessions); (2) the Nordic Musculoskeletal Questionnaire (NMQ), Italian version (28, 29) and (3) The Graded Chronic Pain Scale (GCPS), Italian version (30, 31).

The NMQ explores the prevalence of musculoskeletal complaints, restrictions while performing normal activities and the need for medical attention during the last twelve months and the last seven days, respectively. In addition, this questionnaire contains an illustration of a body map showing the location of the pain area.

The GCPS is composed of questions related to pain intensity and disability, to assess the severity of LBP during the last 6 months prior to completing the questionnaire. It is compiled of seven questions as answers were provided on a scale from 0 (e.g., “no pain” or “no interference/change”) to 10 (e.g., “pain as bad it could be” or “unable to carry on any activity/extreme change”). Pain intensity and disability scores were calculated, and 5 grades of severity were assigned. Grade 0 (pain-free); Grade I (low disability-low intensity); Grade II (low disability-high intensity); Grade III (high disability-moderately limiting); Grade IV (high disability-severely limiting).

### Statistical analysis

Demographic characteristics and training/competition related parameters were presented as number of cases and percentages. The GCPS scores were expressed as mean ± SD. The measures were presented for the total sample and for the subgroups divided by sex and age category (U16 and U20). To assess the potential sex and age category differences as percentages, the Pearson's Chi Square tests were used. An independent sample *t*-test was used to assess the sex and age category differences. For the relationships between the GCPS scores, Spearman's correlational analysis was conducted. Statistical Package for the Social Sciences—IBM SPSS Statistics for Windows, V.28.0 (IBM) was used for all the analysis performed with the statistical significance level set at *p* < 0.05.

## Results

Participants' characteristics including demographic and anthropometric characteristics and sports variables are summarized by sex in Table 1.

**Table 1:** Participants’ characteristics.

Variable	Total (*n* = 180)	Male (*n* = 84)	Female (*n* = 96)
Age	15.79 ± 2.08	16.35 ± 1.90	15.31 ± 2.11
Height (cm)	164.56 ± 9.82	170.14 ± 9.01	159.68 ± 7.69
Weight (kg)	53.22 ± 9.95	58.15 ± 9.77	48.91 ± 7.93
Body mass index (kg/m^2^)	19.49 ± 2.06	19.95 ± 1.95	19.08 ± 2.07
Years of practice	5.64 ± 3.07	5.62 ± 3.16	5.66 ± 3.01
Seasonal competitions	7.94 ± 4.91	7.69 ± 4.81	8.16 ± 5.00
Weekly hours of training	8.20 ± 3.98	8.95 ± 4.23	7.54 ± 3.63
Number of training sessions per week	3.22 ± 0.96	3.37 ± 0.98	3.09 ± 0.93

The NMQ-related results are showed in Table 2. A total of 74.4% of the entire sample of participants reported lower back complaints throughout the past twelve months and only 15.5% during the last seven days. Furthermore, 22.2% of the participants reported that they have been restricted in normal everyday activities within the last 12 months, and 19.4% noted that their lower back complaints needed medical attention throughout the past twelve months. No significant differences were observed between males and females in terms of each NMQ measure.

**Table 2:** Overview of the nordic musculoskeletal questionnaire (NMQ)-based results and differences between sexes and age groups.

NMQ measure	Overall *n* = 180	Male *n* = 84	Female *n* = 96	*Χ*^2^ (df). *p*	U16 *n* = 87	U20 *n* = 93	*Χ*^2^ (df). *p*
Lower back complaints during the last 12 months	134 (74.4%)	63 (75%)	71 (74%)	n.s.	73 (83.9%)	61 (65.6%)	6.99 (1). 0.008
Lower back complaints during the last 7 days	28 (15.5%)	14 (16.7%)	14 (14.6%)	n.s.	8 (9.2%)	20 (21.5%)	4.29 (1). 0.038
Restricted in normal activities during the last 12 months	40 (22.2%)	16 (19%)	24 (25%)	n.s.	11 (12.6%)	24 (25.8%)	4.17 (1). 0.041
Required medical attention during the last 12 months	35 (19.4%)	16 (19%)	19 (19.8%)	n.s.	12 (13.8%)	28 (30.1%)	6.01 (1). 0.014

All NMQ-related measures are expressed as absolute numbers and the percentage proportion on the overall group/subgroups (number of affected climbers/number of climbers per group × 100). Levels of significance for sex are based on Pearson chi-square tests. n.s.: not significant at *p* < 0.05; U-16: under 16 years; U-20: under 20 years.

Climbers competing in U16 category reported significantly higher prevalence of lower back complaints during the last 12 months compared to U20 age group (83.9% and 65.6%, respectively, *p* = 0.008). However, competitors U20 demonstrated higher percentage of lower back complaints within the last seven days in relation to U16 age category climbers (21.5% and 9.2%, respectively, *p* = 0.038). Additionally, athletes competing in the older age category demonstrated greater incidence of restrictions in normal activities throughout the last twelve months than their younger counterparts (25.8% and 12.6%, *p* = 0.041). Similarly, U20 climbers reported higher need for medical attention during the last 12 months than athletes competing in U16 category (30.1% and 13.8%, *p* = 0.014).

The results related to GCPS are showed in Table 3. There were no significant differences between the two age category groups (U16 and U20) in terms of GCPS scores and grades. However, female athletes reported higher Grade II (high intensity-low disability) percentage compared to males (10.4% and 1.2%, respectively, *p* = 0.002).

**Table 3:** Overview of the graded chronic pain scale (GCPS) scores and differences between sexes and categories.

GCPS score	Overall *n* = 180	Male *n* = 84	Female *n* = 96	*z* (df). *p*	U16 *n* = 87	U20 *n* = 93	*Χ*^2^ (df). *p*
Pain intensity	19.6 ± 18.5	17.5 ± 15.3	21.9 ± 20.9	n.s.	18.0 ± 18.3	21.6 ± 18.7	n.s.
Disability score	7.5 ± 13.4	6.4 ± 14.0	8.4 ± 12.9	n.s.	6.3 ± 10.7	8.6 ± 15.5	n.s.
GCPS classification							
Grade 0 pain free	48 (26.7%)	19 (22.6%)	29 (30.2%)	n.s.	29 (33.3%)	19 (20.4%)	n.s.
Grade I Low intensity-low disability	113 (62.8%)	61 (72.6%)	52 (54.2%)	n.s.	52 (59.8%)	64 (68.8%)	n.s.
Grade II High intensity-low disability	13 (6.1%)	1 (1.2%)	12 (10.4%)	9.31 (1). 0.002	5 (5.75%)	8 (8.6%)	n.s.
Grade III High disability-moderately limiting	2 (2.8%)	1 (1.2%)	1 (4.2%)	n.s.	1 (1.15%)	1 (1.1%)	n.s.
Grade IV High disability-severely limiting	1 (0.6%)	1 (1.2%)	0 (0%)	—	0 (0%)	1 (1.1%)	—

GCPS scores are expressed as mean ± SD. GCPS classifications are expressed as absolute numbers and the percentage proportion of the overall group/subgroups (number of affected climbers/number of climbers per group×100). Levels of significance for sex and category differences are based on Mann-Whitney tests and Pearson chi-square tests, respectively.

n.s.: not significant at *p* < 0.05; U-16: under 16 years; U-20: under 20 years.

No significant correlation was observed between GCPS and sport practice-related questions (years of practice, seasonal competitions, weekly hours of training, training sessions per week), neither in the case of stratification of training attributes nor in stratification by gender.

## Discussion

This study aimed to determine the prevalence and severity of LBP with respect to sex and age category. We found a relatively high prevalence of LBP in young climbers, mainly classified as low-intensity or pain-free. Climbers did not differ in LBP prevalence according to sex. Concerning age groups, younger climbers reported significantly higher prevalence of lower back complaints during the last 12 months, while older climbers demonstrated higher percentage of lower back complaints within the last seven days, greater incidence of restrictions in normal activities throughout the last twelve months, and higher need for medical attention during the last 12 months. The relationship between lower back complaints severity and training attributes was not significant for the total sample nor sex- or age-stratified.

### The prevalence of low back pain in adolescent climbers

We observed a relatively high prevalence of LBP during the last 12 months in Italian young climbers. However, concerning the GCPS classification, most of the LBP was classified as pain-free and low intensity, and low disability, which means that LBP was not that severe and intense to affect climbers' regular competing and training sessions.

We observed low LBP severity, suggesting low adverse back loading, which is in agreement with the relatively low percentage of spine injuries reported in the literature. Specifically, several studies investigated climbing-related chronic injuries by observing the whole body, which includes low back and LBP complaints. Specifically, a study on 667 climbers aged 26–40 years recorded that 385 climbers had chronic injuries, from which only 11 climbers (2.9%) had LBP (3). Similarly, a study on 836 climbers aged 34.1 ± 11.1 years noted that only 11 cases (1.2%) of LBP have been reported, out of 911 total injuries (32). In a relatively recent comprehensive review of climbing injuries, it was reported that injuries of the spine account for 1.9%–7.1% of all climbing injuries (2). The study which focused on investigating LBP in climbers aged 29 ± 7 years found that 26% of included climbers reported mild LBP (33). It must be emphasized that the previously mentioned study used the Oswestry Low Back Pain Disability Index (which demonstrated good validity and reliability) categorizing LBP intensity into mild, moderate, and severe, which means that the reported LBP led to light or no restrictions at all (34).

Noteworthy, the prevalence and severity of LBP in other sports is significantly higher than in climbers. Precisely, in a study on 1,114 elite German athletes aged 20.9 ± 4.8 years, the lifetime prevalence of LBP was 89%, prevalence during the last 12 months was 81%, and prevalence during the last 3 months was 68% (16). It has to be noted that the prevalence of LBP differed according to sports disciplines, with athletes involved in waterpolo (100%), fencing (100%), rowing (96.4%), gymnastics (93.8%), and dance (95.5%) having the highest rates of LBP (16, 17). Also, a study on athletes involved in repetitive overhead activities (i.e., volleyball, handball, tennis) reported that the lifetime prevalence of LBP was 85%, and the prevalence during one year was 75% (35). On the other side, the prevalence of LBP during one year was reported to be lower (50%–65%) in different sports among elite athletes, for instance in cross-country skiers and rowers (36, 37).

Considering youth athletes, LBP is also a common complaint as 10%–15% of youth athletes report LBP, but this percentage varies according to sports (e.g., 27% of college football, and 86% of rhythmic gymnasts) (21). Adolescent athletes involved in sports that have repetitive extension, flexion, and rotation of the spine (e.g., gymnastics, soccer, dance) are the most susceptible to injuries in the lower back (17). Furthermore, a study on adolescent alpine skiers aged 15–18 years, that used a similar methodology as our study, reported that 80.3% of skiers suffered LBP during the last 12 months, and 50.7% during the last 7 days, which is a higher prevalence than in our study (38). Moreover, LBP among young alpine skiers was in 21.8% of cases classified as high intensity/low disability, which is of significantly higher prevalence than in our study (6%). Also, the characteristic pain intensity was higher in alpine skiers compared to climbers (37.53 ± 18.00 and 19.60 ± 18.50, respectively), so was the disability score (13.27 ± 14.59 and 7.50 ± 13.40) (36).

Therefore, we could speculate that young climbers display lower LBP complaints compared to athletes participating in other sports, as climbing might facilitate the improvement in motor control and coordination (39), and the strength development without a functional overload. Due to these factors, climbing might be beneficial for preventing and treating LBP. Previous studies focused on investigating the positive effects of climbing (i.e., therapeutic climbing) on LBP, but mostly in non-athletic population (39). Indeed, the impact of climbing on patients with LBP has been evaluated on patients divided into climbing groups, who practiced climbing exercises for 8 weeks, and the control group, who did not practice climbing (24). Patients in the climbing group displayed a reduction in the size of disc protrusion and a reduction in overall back pain compared to the control group (24). Authors of that study theorized that climbing offers closed-chain muscle exercise which improves muscle control and posture, resulting in less pain (24). However, studies that investigated the effects of therapeutic climbing on back pain included a non-climbing population and activity was at low intensity. Thus, prospective studies on competitive climbers are needed to prove the hypothesis of the beneficial effects of climbing on LBP.

### Sex differences in the prevalence of low back pain and the relationship between lower back complaints severity and training attributes

The results of our study did not show sex differences in the prevalence of LBP. Also, there were no sex-specific associations between LBP and training attributes. Previous studies reported controversial results regarding the relationship between LBP and sex in athletes (16). Some studies reported that females are more likely to report LBP (40, 41), while other studies found higher rates of LBP for males (42, 43). Sex differences in LBP and other musculoskeletal injuries in athletes are influenced by numerous factors. Specifically, males might be exposed to higher loads in some sports disciplines because they have higher training volume and higher loads during strength training, or they might have different rules (e.g., game duration) (16). Also, females have a menstrual cycle that sometimes prevents them from partaking in training sessions and reduces overall training volume (44). Additionally, a study on adolescents aged 11–19 years with chronic pain conditions noted differences in pain tolerance between sexes (i.e., females reported lower pain threshold than males) and pain-coping strategies (i.e., females used more social support while males engaged in behavioural distraction) (45).

Overall, our results showed no sex differences in LBP severity and no associations with training attributes which could be explained by the specificity of sport. A recent study on a similar cohort of young climbers investigated gender differences in generic- (countermovement and squat jump, grip strength) and specific-fitness test (power slap test and Draga foot lift) of youth climbers and found no differences in climbing-specific-fitness profiles comparing males and females, while there were sex differences in the generic-fitness profile (46). Authors suggested that climbing requires specific abilities similar in males and females, which could be the reason why we did not detect differences in LBP between sexes (46). This theory could be further confirmed by a review study that investigated injury risk factors in climbers which reported that six studies found no differences in injury risk between males and females, accounting for whole-body injuries (6). Thus, we could hypothesize that, due to the specificity of climbing and similar loads, males and females do not differ in the prevalence and intensity of LBP.

### Age differences in the prevalence of low back pain and the relationship between lower back complaints severity and training attributes

Our results showed age differences in NMQ low back complaints among youth competitive climbers, with older climbers reporting a higher incidence of LBP during the last 7 days and a greater occurrence of seeking medical attention than younger climbers. Similar to the results of our study, a study on Canadian youth climbers aged 11–19 years reported that adolescent climbers (15–19 years) have 11.3 times greater risk of injuries compared to younger climbers aged 11–14 years (9). Moreover, a study on a large sample of young athletes involved in combat sports, game sports, explosive strength sports, and endurance sports found that younger athletes (11–13 years) had 2%–4% of LBP while the prevalence increased to 12%–20% in older athletes (14–17 years) (47). Thus, from the results of the NMQ, it could be concluded that older climbers are more predisposed to experience LBP. What is somewhat surprising and confusing is the result that younger climbers reported a higher incidence of LBP during the last 12 months. We could speculate that younger climbers were less accurate in reporting results regarding the 12-month recall period.

Our results noted that climbers did not differ in LBP severity according to age groups and there were no LBP associations with training attributes. These results could be explained in light of similar performance levels in different age groups (48). In our study, younger (U16) climbers had only one year of training experience less than older (U20) climbers (5.05 years and 6.19 years of climbing practice, respectively). Thus, the small difference in the years involved in climbing practice and exposure to training-induced musculoskeletal stress could be the reason for not recording differences in LBP severity between younger and older adolescents.

## Limitations and strengths

The main limitation of the study is its cross-sectional design, unable to determine causality. Moreover, the limitation is that injuries were self-reported and retrospective, which could potentially lead to recall bias. We were also unable to categorize the incidence of LBP according to a specific climbing discipline, this is due to the fact that climbers in our study competed in all the three disciplines, as required by the rules of the climbing federation for the U16 and U20 age categories. An additional limitation is that it was not possible to determine the climbing level expressed through the highest climbed grade (both sport climbing routes and boulders). This is due to youth climbers not frequently (or at all) climbing outdoors where there are graded climbing routes or boulders, as they most commonly practice indoors.

The main strength of the study is related to the novelty of the information collected. To date, we still know little about the prevalence of LBP in young climbers; investigating musculoskeletal complaints in adolescent athletes could lead to detecting injury risks, so that appropriate preventive actions and effective treatment programs can be planned.

### Practical implications

Young climbers in this study reported lower LBP complaints compared to young athletes participating in other sports, this could be due to a combination of specific factors, such as motor control and coordination and muscle strength improvement. It might therefore appear that climbing might be beneficial for preventing and treating LBP. Whether this can be true for recreational climbing, on the other hand it is important to consider that the style of climbing competitions has been changed profoundly in recent years into more dynamic and physically demanding. This trend might lead into increased prevalence and severity of musculoskeletal conditions, and within these of LBP, in athletes. Therefore, the results of this study can be used to disseminate the message that it is important to adopt preventive strategies for LBP, which should be regularly implemented into training routine. Also, prospective evaluation of LBP should be applied in future research and coaching practice with the aim of monitoring LBP and preventing more serious complaints and injuries.

## Conclusion

The results of this study showed that there was a relatively high prevalence of LBP in young climbers, but it was mainly classified as low-intensity or pain-free. Furthermore, climbers did not differ in LBP prevalence according to sex and a significant but small difference in age groups was recorded. According to the results, it could be theorized that climbing, including competitive climbing, may not be particularly harmful to the lower back. However, the results should be interpreted with caution and no strict conclusions can be drawn due to the cross-sectional nature of the study. Also, considering that the climbing style is changing rapidly to more dynamic movement patterns, especially among competitive climbers, LBP could be expected to become more common than before. Thus, future studies should prospectively monitor the influence of climbing on LBP in youth climbers. This way, coaches would be able to identify risk factors for LBP occurrence and prevent this common health problem.

## Data Availability

The raw data supporting the conclusions of this article will be made available by the authors, without undue reservation.
